# The Epidemiological Modelling of Major Depressive Disorder: Application for the Global Burden of Disease Study 2010

**DOI:** 10.1371/journal.pone.0069637

**Published:** 2013-07-29

**Authors:** Alize J. Ferrari, Fiona J. Charlson, Rosana E. Norman, Abraham D. Flaxman, Scott B. Patten, Theo Vos, Harvey A. Whiteford

**Affiliations:** 1 University of Queensland, School of Population Health, Herston, Queensland, Australia; 2 Queensland Centre for Mental Health Research, Wacol, Queensland, Australia; 3 University of Queensland, Queensland Children's Medical Research Institute, Herston, Queensland, Australia; 4 University of Washington, Institute for Health Metrics and Evaluation, Seattle, Washington, United States of America; 5 University of Calgary, Department of Community Health Sciences, Calgary, Canada; University of Iowa Hospitals & Clinics, United States of America

## Abstract

**Background:**

Although the detrimental impact of major depressive disorder (MDD) at the individual level has been described, its global epidemiology remains unclear given limitations in the data. Here we present the modelled epidemiological profile of MDD dealing with heterogeneity in the data, enforcing internal consistency between epidemiological parameters and making estimates for world regions with no empirical data. These estimates were used to quantify the burden of MDD for the Global Burden of Disease Study 2010 (GBD 2010).

**Method:**

Analyses drew on data from our existing literature review of the epidemiology of MDD. DisMod-MR, the latest version of the generic disease modelling system redesigned as a Bayesian meta-regression tool, derived prevalence by age, year and sex for 21 regions. Prior epidemiological knowledge, study- and country-level covariates adjusted sub-optimal raw data.

**Results:**

There were over 298 million cases of MDD globally at any point in time in 2010, with the highest proportion of cases occurring between 25 and 34 years. Global point prevalence was very similar across time (4.4% (95% uncertainty: 4.2–4.7%) in 1990, 4.4% (4.1–4.7%) in 2005 and 2010), but higher in females (5.5% (5.0–6.0%) compared to males (3.2% (3.0–3.6%) in 2010. Regions in conflict had higher prevalence than those with no conflict. The annual incidence of an episode of MDD followed a similar age and regional pattern to prevalence but was about one and a half times higher, consistent with an average duration of 37.7 weeks.

**Conclusion:**

We were able to integrate available data, including those from high quality surveys and sub-optimal studies, into a model adjusting for known methodological sources of heterogeneity. We were also able to estimate the epidemiology of MDD in regions with no available data. This informed GBD 2010 and the public health field, with a clearer understanding of the global distribution of MDD.

## Introduction

Quantitative summaries of disease epidemiology are essential inputs to generating health indicators such as burden of disease estimates. They have also made significant contributions to health policy, service planning, and funding priorities in public-health [1]–[3]. That said, epidemiological data can be costly and difficult to assemble. As a result global data are limited and sometimes unreliable, in which case supplementary measures to accurately compile the data are required.

The Global Burden of Disease (GBD) 1990 study and its update in 2000 quantified burden in terms of disability-adjusted life years (DALYs) which are the sum of years of life lived with disability (YLD) and years of life lost due to premature mortality (YLL) [4], [5]. In 1990, depressive disorders were the 4th leading cause of burden [4]. In 2000, they were the 3^rd^ leading cause of burden as well as the leading cause of disability [6]. This has made the estimation of burden for depressive disorders a critical component of the GBD 2010 study. GBD 2010 is a comprehensive re-assessment of disease burden and draws on a wide range of data sources and expertise to quantify burden for 291 diseases and injuries, 21 world regions and the years 1990, 2005 and 2010. Main findings from this study were published in 2012 in a series of publications [7]–[13].

The GBD 2010 mental disorders research group (see: http://www.globalburden.com.au/) oversaw the burden quantification process for 20 mental disorders, including major depressive disorder (MDD) and dysthymia. For each mental disorder, this involved: (1) conducting a literature review of the disorder's epidemiology; (2) evaluating the extracted data in a disease model; and (3) producing estimates of prevalence for calculating disease burden [9], [11]. A major point of difference of GBD 2010 from previous versions is that results were presented without discounting, without the previously used age weights and with prevalent rather than incident YLDs [11]. This paper follows our literature review of the raw global epidemiological data for MDD [14], representing step 1 of the burden calculation process. Here, we present an integrated and complete epidemiological model of MDD (step 2). The epidemiological review and modelling of dysthymia is being reported separately [15].

For GBD purposes, epidemiological data on prevalence, incidence, remission, duration and excess mortality are required [11]. Summarising these parameters for MDD: (1) there are more data available for prevalence than for other parameters; (2) there are sparse data from low and middle income countries; and (3) there is considerable between-study variability in the epidemiology of MDD [6], [14], [16]–[20]. This epidemiological variability may be an artefact of differences in data collection and assessment or, alternatively, due to real differences in the disorder's epidemiology [21]–[23]. The aim is to correct for the former and to retain the latter in order to present an accurate epidemiological profile of the burden of MDD.

Existing reviews of the global prevalence of MDD suggest that the 12-month prevalence ranges between 0.8% and 5.8% [18] or between 2.2% and 10.4% [16], depending on study methodology and sampling. Given that GBD focuses on capturing people who are experiencing disability within the year of interest, period prevalence is not the ideal measure for quantifying disease burden [11]. Our review estimated that the global point (defined as current or past-month) prevalence of MDD was 4.7% (4.4%–5.0%) ranging from 3.7% (3.1%–4.3%) in North America to 8.6% (5.2%–14.0%) in South Asia, a region which included prevalence from countries in conflict. Study methodology and geographic location explained 57.7% of the variability in prevalence, noting that lack of data for certain parts of the world limited findings [14]. Our pooled estimate of annual incidence derived from studies identified in the systematic review was 3.0% (2.4%–3.8%). As the estimated average duration of a major depressive episode is less than a year [24], it is clear that the prevalence and incidence findings were ‘inconsistent’ as logically, incidence of MDD episodes should be higher than prevalence.

**Figure 1:**
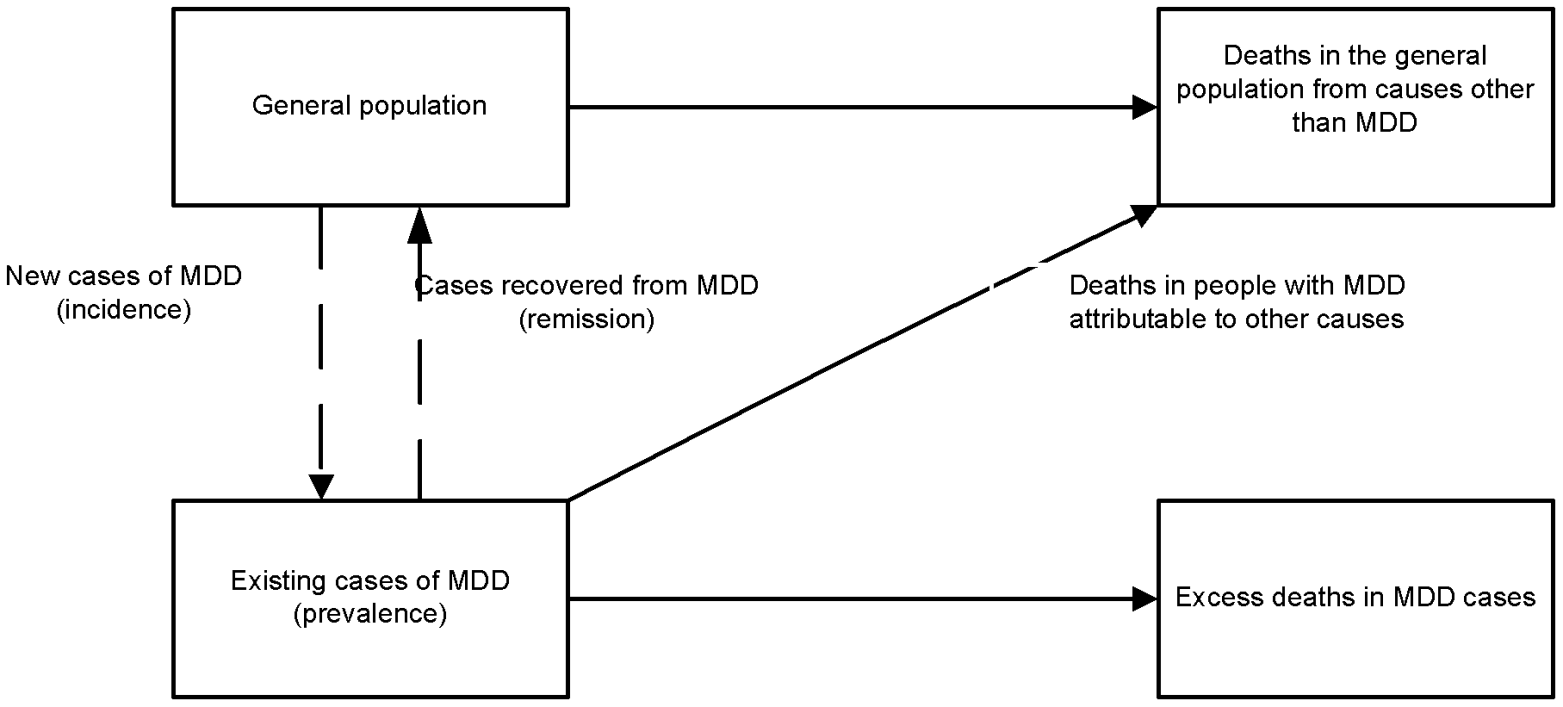
Incidence-prevalence-mortality model. Illustrates the generic relationship between epidemiological parameters used by DisMod-MR to model epidemiological data.

Internal consistency can be achieved by making use of an incidence-prevalence-mortality model (Figure 1) to check for, and force consistency between epidemiological parameters. This is when final prevalence, incidence, duration and excess-mortality estimates simultaneously adhere to the generic relationships in the incidence-prevalence-mortality model for a single time, place, and sex [11], [25], [26]. More specifically, people in the general population are at risk of becoming ill and after incidence, become prevalent cases of MDD. They are then at risk of dying as a result of MDD and contributing to the cause-specific mortality rate or they may recover, contributing to the remission rate. People with and without MDD are also at risk of dying from other causes. Internal consistency is met if there is a corresponding incident case for every prevalent case of MDD; and if the total number of prevalent cases for MDD reflects not only prevalent and incident cases but also individuals that have died (as a result of MDD or other causes) and individuals that have recovered from MDD.

Supplementing this model with expert knowledge also helps address other limitations in the empirical data. For instance, identifying relevant covariates from study design and methodology (e.g. prevalence period) helps to adjust sub-optimal data. Making predictions based on the raw data and identifying relevant ecological covariates (e.g. conflict status) enables us to estimate data for parameters and world regions with no available data. Excluding these parts of the world from GBD estimations would assume no burden from those countries hence exclude them from the global priority setting exercises intended for GBD 2010 findings. Conscious of the importance of accurately representing all world regions in global health agendas, the GBD 2010 approach was not only to predict epidemiological data for parts of the world with missing data but to also ensure that the resulting uncertainty around these predicted estimates was incorporated into final burden results.

In this paper we present an internally consistent epidemiological profile of MDD generated by DisMod-MR, a Bayesian meta-regression tool [11], [26] that predicts epidemiological data for parameters and parts of the world with no raw data and accommodates known methodological and ecological determinants of MDD through the use of covariates. Aside from informing GBD 2010 burden estimates, this work contributes to the wider MDD literature by providing a more accurate and complete depiction of the global distribution of MDD.

## Methods

### Case definition

The Diagnostic and Statistical Manual of Mental Disorders (DSM-IV-TR) characterises MDD by one or more major depressive episodes, lasting for at least 2 weeks [27], a definition resembling that of recurrent depressive disorder in the International Classification of Diseases (ICD-10) [28]. A major depressive episode involves symptoms of depressed mood and/or loss of interest or pleasure in all or almost all activities occurring most of the day and nearly every day. Consistent with the methodology proposed by Vos and collaborators [29], [30] as well as Ustun and collaborators [31], we modelled MDD as an episodic disorder, with the incidence and average length (i.e. duration) of an episode specified. We also incorporated prevalence estimates of depression not otherwise specified (NOS). This was in response to literature suggesting that MDD is often coded as depression NOS in non western regions because DSM/ICD diagnostic criteria are less sensitive to non-western presentations of the disorder [32]–[34].

### Search strategy

Estimates of prevalence, incidence, duration and excess mortality were searched for in a systematic review of the literature. This methodology has been outlined in greater detail elsewhere [14], [24], [35] with the PRISMA checklist and flowchart [36] summarised in supporting text S1. In summary, electronic databases Medline, PsycInfo and EMBASE were searched from 1980 onwards and studies were included if prevalence, incidence, duration and/or excess mortality of MDD were reported and if they were representative of the community, region or country. DSM or ICD diagnostic categorisations were required although if studies used symptom scales that broadly mapped to DSM/ICD thresholds, these were also included for prevalence due to lack of data in low to middle income regions. For prevalence we also required past year or point estimates. Even though point prevalence is the more representative measure for GBD purposes as it measures actual disability, 12–month prevalence was accepted to maximise inclusion. Lifetime estimates were excluded as they are most susceptible to recall bias [37]–[40]. Given these allowances made to the inclusion criteria, we then looked at ways to adjust sub-optimal estimates (e.g. estimates derived from symptom scales and based on 12-month prevalence) towards optimal estimates (e.g. estimates derived from diagnostic instrument and based on point prevalence) to minimise the methodological heterogeneity in the dataset (see covariates section). For incidence we used hazard rates, with person years of follow-up in the denominator; for duration we used estimates based on follow-up studies reporting the natural history of MDD in community samples; for excess mortality we used relative risks (RR; i.e. deaths in people with MDD compared to people without MDD) or standardised mortality ratios (SMR; i.e. deaths in people with MDD compared to deaths in the general population).

Epidemiological data were extracted into a Microsoft Excel spreadsheet. Along with information pertaining to the study methodology, design, parameter type and value, an estimate of uncertainty (standard error (SE) or 95% confidence interval) was extracted if reported. If not reported, uncertainty was calculated using SE = √2.1*(P*(1–P)/N) where P is the proportion of cases reported and 2.1 is an average design effect calculated using 110 design effects from the GBD Mental Disorders Research Group's affective disorders dataset. N is the age- and sex-specific denominator which, if not reported, was estimated using United Nation's country-, sex-, age- and year-specific population size to apportion the study sample size across age and sex categories [41].

The country in which each study was conducted was coded according to the 21 world regions (see: http://www.globalburden.com.au/project-description) used for GBD 2010. This regional grouping was based on broad geographic regions or continents where each region comprised of no fewer than two countries, grouped according to country-specific child/adult mortality levels and major causes of death [9], [11]. Seven ‘super-regions’ were also defined which grouped regions according to cause of death patterns (Super-region 0: high income regions-Asia Pacific High Income, Australasia, Western Europe, Latin America South and North America High Income; Super-region 1: Central and Eastern Europe and Central Asia; Super-region 2: West, East, Central and Southern Sub-Saharan Africa; Super-region 3: North Africa and Middle East; Super-region 4: Asia South; Super-region 5: East and Southeast Asia and Oceania; Super-region 6: Central, Andean and Tropical Latin America and Caribbean). The aim of this was to categorise countries into regions and regions into super regions approximating more epidemiologically homogeneous groups. These were used to guide the estimation of missing data informed by data from surrounding countries and/or regions [9], [11].

### Empirical data

**Figure 2:**
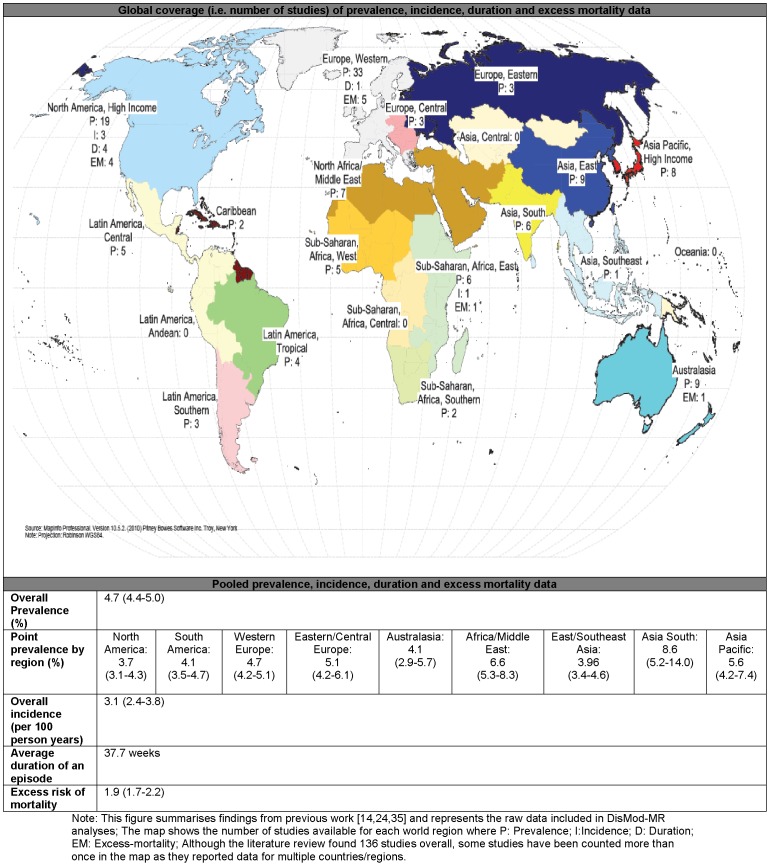
Summary of the raw data on prevalence (P), incidence (I), duration (D) and excess mortality (EM) of MDD. Summarises the available epidemiological data used as inputs in the DisMod-MR modelling of MDD.

The systematic literature review identified 136 relevant studies covering 17 GBD world regions. Epidemiological estimates were reported for males, females and/or persons, across broad and/or specific age groups. Sex- and age-specific estimates were preferable. Figure 2 summarises the raw epidemiological data used as inputs in the disease modelling process. A more comprehensive summary of the included studies has been reported elsewhere [14], [24], [35].

For prevalence, we found 116 studies (556 data points) from 53 countries and 17 regions. After further consideration, 3 prevalence studies were excluded as outliers in the dataset [42]–[44]. Estimates from these studies were well over 2 times higher than other estimates from the same country and/or region, and stood out as outliers in the initial stages of the modelling process. This reduced the prevalence dataset to 113 studies (544 data points). For incidence we found 4 studies (19 data points) on annual incidence from 3 countries and 2 regions [14]. For duration, we found 4 studies from USA [45]–[48] and 1 study from the Netherlands [49] reporting a median duration of between 6 to 12 weeks. We replicated the methodology used by Vos and collaborators to estimate an average duration from a best fit curve between the data points available from all 5 studies reporting on time to end of an episode [24]. For excess-mortality we found 11 studies (14 data points) from 7 countries and 4 regions as compiled by Baxter and collaborators [35].

### Analyses

DisMod-MR was used to model the epidemiology of MDD. DisMod-MR is the latest iteration of the generic disease modelling system [25] but redesigned as a Bayesian meta-regression tool [11], [26]. The Bayesian approach is one of several interpretations of statistical probability in which existing data is used to inform the probability of a given hypothesis i.e. the data is considered as fixed and the hypothesis as random. This is different to the frequentist approach for instance which quantifies the probability (or frequency) of the data given the hypothesis i.e. the data is considered random and the hypothesis fixed [50]; A meta-regression can be understood as an extension of a meta-analysis whereby data from different studies are pooled into a weighted average, adjusting for sources of variability between studies [51]. DisMod-MR has the capability to combine epidemiological data from multiple sources, reconcile data that are inconsistent and forecast data for regions and parameters with no or little data. It applies a negative-binomial model of disease prevalence, incidence, remission, and case-fatality rates and fits models with a randomized Markov-Chain Monte Carlo algorithm [11], [26]. Non-fatal burden estimates for all disorders in GBD 2010 were calculated using DisMod-MR with the exception of a few conditions for which a customised model had to be created [11].

DisMod-MR works in two stages. At stage 1 it pools raw data for each parameter while incorporating prior expert knowledge of the disease (based on empirical evidence and expert knowledge of the distribution of MDD in the population). In the absence of sufficient data to show age-pattern variation, DisMod-MR imposes a common age pattern based on evaluating age-specific input data available for the disorder. This stage also includes a first consistency check at the global level. At stage 2, DisMod-MR simultaneously integrates the input data from all parameters as well as the output from stage 1 to derive internally consistent epidemiological estimates for 187 countries, 21 GBD world regions for 1990, 2005 and 2010, carrying forward uncertainty from primary data sources [11], [26]. These 3 time periods were chosen to enable analysis of time trends and enable comparisons between different time periods using the same methodology. It would not be possible to compare time trends using GBD 1990 estimates from the original study as methodology is different. If the period of data collection was before and including 1997 (the midpoint between 1990 and 2005) then those studies contributed to the 1990 estimates. Studies with data collected after 1997 contributed to the 2005 and 2010 estimates. Although the year 2000 would have also been a sensible alternative to categorise estimates as it is the midpoint between 1990 and 2010, there was insufficient data to detect any difference in the current findings if the latter option had been used. Where relevant, year-specific country-level covariates informed the difference between 1990, 2005 and 2010 estimates. Regions without primary data borrowed strength from other regions in a super-region through random effects. If a whole super-region had no data, epidemiological estimates defaulted to the global average unless country-level covariates were specified [9], [11].

### Adjustments to the data

As per the Bayesian approach, a range of adjustments were implemented during the modelling phase to account for prior knowledge of disease patterns. A mimimum age of onset of 3 years was selected based on literature and expert advice suggesting that despite difficulties in diagnosing early childhood depression, cases of MDD manifest as early as 3 years [52]. Adjustments were also used to supplement gaps in the raw data. After running sensitivity analyses with and without the incidence data included in the DisMod-MR model (see results section), it was deemed necessary to exclude the few data points showing low rates of MDD incidence in the population. MDD was modelled as an episodic disorder (as per how it's defined in DSM-IV-TR)/ICD-10), as such we required data on the incidence and duration of a major depressive episode in the DisMod-MR modelling of MDD. In our review of the literature, we found that the average duration of a major depressive episode was less than a year. Based on this, we would expect the incidence of a major depressive episode to be higher than the prevalence of MDD. In the four studies we had available for incidence, new major depressive episodes in people with previous episodes were either excluded at baseline [53], [54], discussed but not included in the final incidence estimate [55], or alternatively reported but limited to a narrow teenage sample where the incidence of new episodes comes close to total incidence (previous plus current episodes) [56].This meant that for our purposes, incidence was underestimated and ‘inconsistent’ with prevalence and duration data. Given this limitation, we excluded the few incidence estimates available and instead, allowed DisMod-MR to predict incidence based on the data from all other parameters. The estimate of average duration was applied equally to all regions, sex and years given that there were only 5 follow-up studies available with information on time to end of an episode and none of those 5 studies found statistically significant sex differences in episode duration.

### Covariates

#### Study-level covariates

The prevalence dataset included estimates of point and past-year prevalence based on varying survey instruments, response rates and sample coverage [14]. Study-level covariates were applied to adjust sub-optimal estimates to the desired level of each of these variables (Table 1). Our meta-regression of the raw prevalence data outside of DisMod-MR [14] guided the selection of these study-level covariates.

**Table 1:** Study-level covariates used in the statistical modelling of MDD.

Study-level covariates		
Covariate	Gold-standard	Rationale
Prevalence type	Point prevalence	GBD methodology requires point rather than 12-month prevalence. Given their structure, diagnostic interviews capturing 12-month prevalence may also be insensitive to past major depressive episodes, leading to the underestimation of prevalence.
Survey intrument	Instruments using DSM/ICD diagnostic thresholds	Symptom scales are likely to over-estimate prevalence relative to diagnostic instruments using DSM/ICD thresholds [14]. Prevalence estimates based on symptom scales are adjusted to the level of those using DSM/ICD diagnostic thresholds
Sample coverage:	National coverage	This study level covariate adjusts prevalence ascertained from a local community sample to the level of prevalence from a more representative regional or national sample.
Study response rate	response rate ≥60%	This study level covariate adjusts prevalence from samples with poor response rate (<60%) to the level of those with better response rate (≥60%).

**Country-level covariates**		
**Covariate**	-	**Definition**
Conflict	-	The natural log of mortality due to war or conflict in any country and year.
Post conflict	-	The natural log of mortality due to war or conflict in the past ten years, in any country and year.

#### Country-level covariates

Country-level covariates guided the DisMod-MR estimation of prevalence, particularly in the prediction of missing data. As described in previous work [57]–[60], conflict status is associated with an increase in the incidence (and therefore prevalence) of MDD. We also found evidence for this while comparing pooled prevalence across regions including countries in current or past conflict [14]. To improve the predictive power of the model for regions with no data, we included conflict and post conflict covariates in the modelling of prevalence. These covariates used the natural log of GBD 2010 mortality rates due to war or conflict in any country and year (Table 1).

## Results

To demonstrate the effect of the DisMod-MR modelling process on the epidemiological data, the first section of results compares the input data to the final DisMod-MR output. In spite of being limited by data on the incidence, duration, and excess-mortality of MDD, we discuss the DisMod-MR output for all parameters here in the interest of illustrating internal consistency. The next section focuses exclusively on the prevalence output given that the majority of our data was for prevalence and GBD 2010 calculated prevalent YLDs. More information on the DisMod-MR output can be obtained by contacting the corresponding author (AJF).

### Comparing data points with final DisMod-MR output

**Figure 3:**
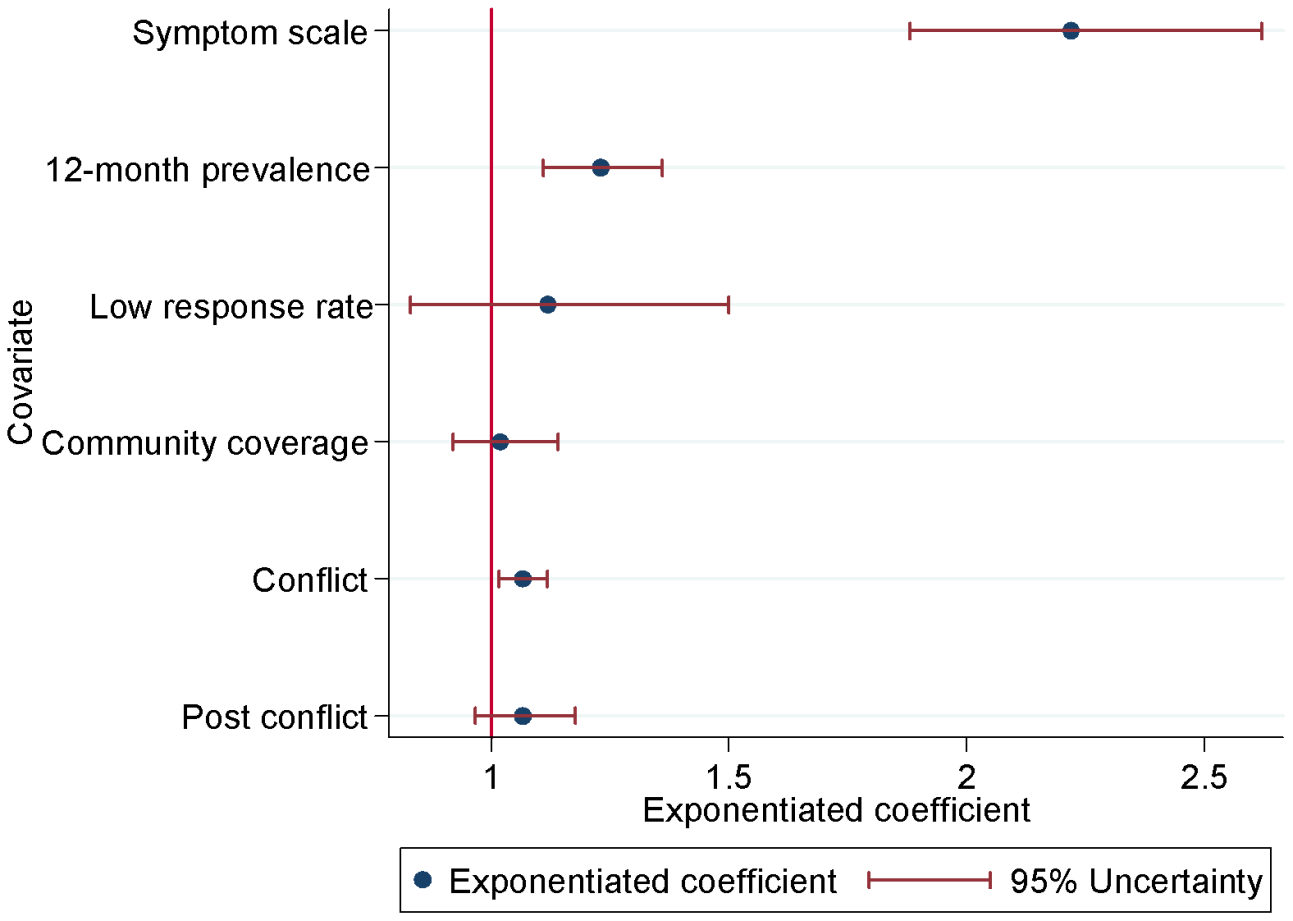
Country- and study-level covariate adjustments for MDD. Illustrates the effect of the covariates used in the DisMod-MR modelling of prevalence data.

Figure 3 shows the adjustments made to prevalence based on the effect of covariates. Study-level covariates for data points of past-year prevalence and using symptom scales had statistically significant positive values. Those data points were adjusted downwards to reflect an equivalent value if the studies would have measured point prevalence, using formal diagnostic instruments. The study response rate and community coverage covariates did not significantly impact on prevalence. There was however a positive effect of conflict whereby prevalence from countries in current conflict was higher than prevalence from countries in no conflict. This effect guided the prediction of prevalence for regions with missing data.

**Figure 4:**
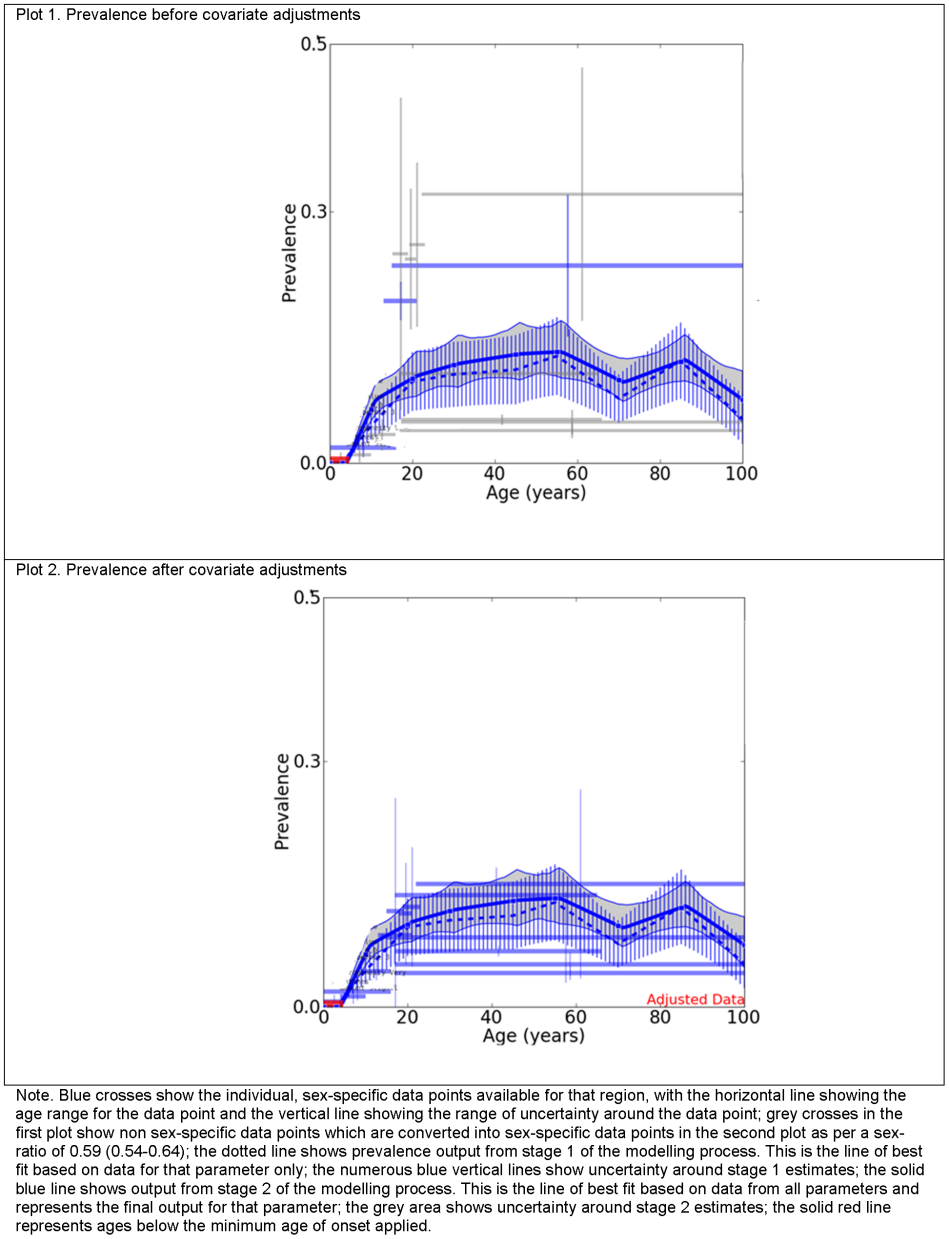
Prevalence of MDD before and after covariate adjustments for females from North Africa/Middle East, 2010. Compares the raw prevalence estimates to the final pooled prevalence output generated by DisMod-MR for females from North Africa/Middle East, for 2010.

**Figure 5:**
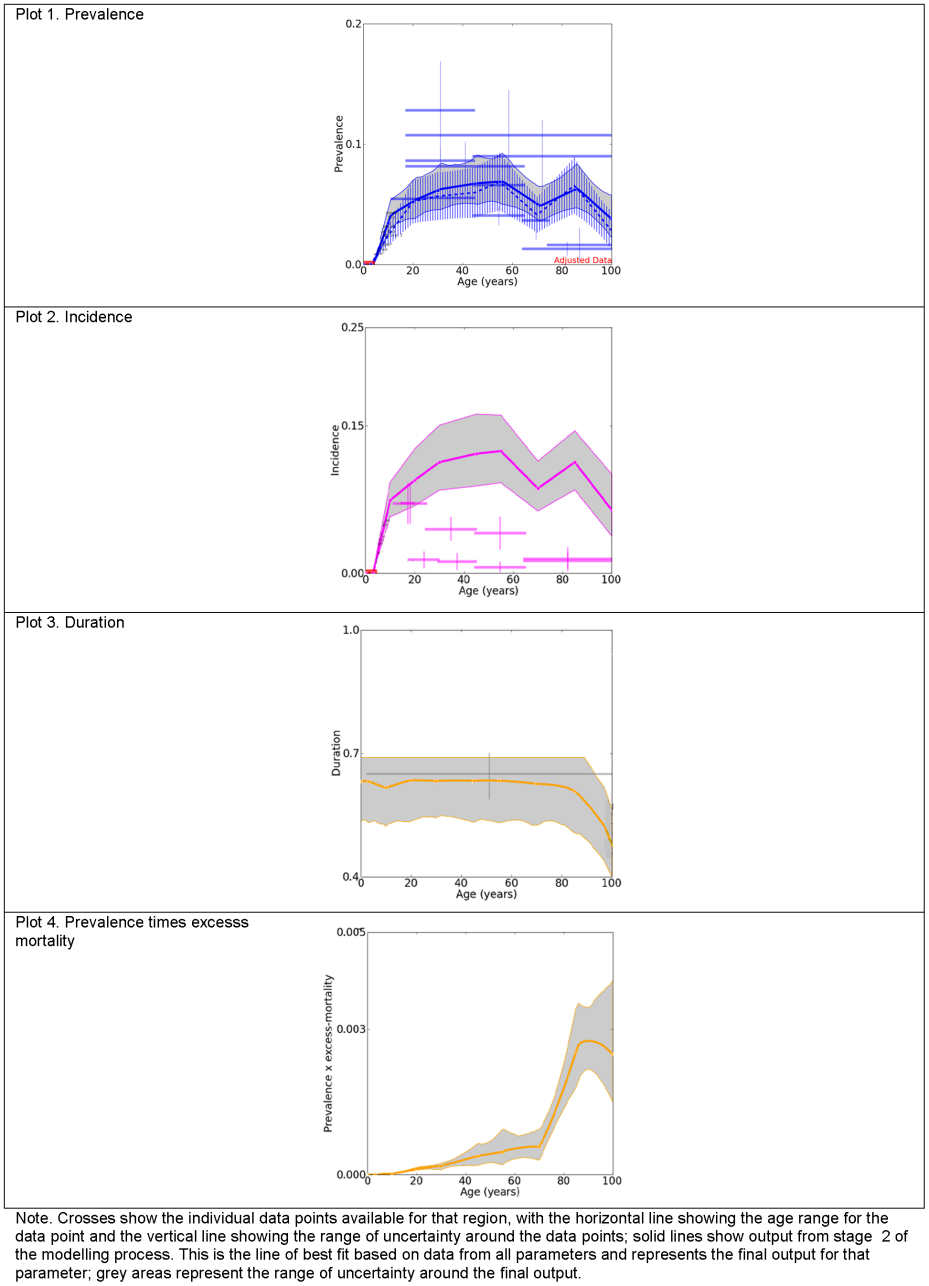
Prevalence, incidence, duration and excess mortality of MDD in females from North America, High income, 2010. Compares the raw prevalence, incidence, duration and excess mortality estimates to the final pooled prevalence, incidence, duration and excess mortality output generated by DisMod-MR for females from North America, High income, for 2010.

Figures 4 and 5 further illustrate the adjustments applied to the input data by summarising the input data and DisMod-MR output for females in 2010 from North Africa/Middle East (figure 4) and North America, High income (figure 5), regions for which we had few and considerable data points, respectively. Each plot in figure 4, shows the minimum age of onset of 3 years (solid red line), the prevalence data points (blue crosses) and the final pooled prevalence output (solid blue line) before (plot 1) and after (plot 2) they were adjusted by study-level covariates. The difference between the two plots reflects adjustments made by study level covariates. Note how the prevalence data points were adjusted downwards (from plot 1 to plot 2) to reflect an equivalent value if the studies would have measured point prevalence, using formal diagnostic instruments.

The first plot in figure 5 shows the prevalence data and the second plot shows the incidence data with their respective pooled DisMod-MR output. As previously explained, incidence data points (pink crosses) in plot 2 were not included in the modelling process. However, it is worth noting here how much lower they were in comparison to the incidence calculated by DisMod-MR (solid pink line), using data from all other parameters. In dealing with the previously noted inconsistency between incidence, prevalence and duration data, the final incidence output was also greater than the final prevalence output in plot 1. Incidence was greater by a fixed amount as we applied the same estimate of average duration across all regions, sex, age and year. The third plot from figure 5 shows the single duration data point used (grey cross). The last plot shows prevalence by excess mortality which represents the mortality rate at the population level due to the (all-cause) excess mortality experienced by people with MDD.

### Final prevalence output

**Figure 6:**
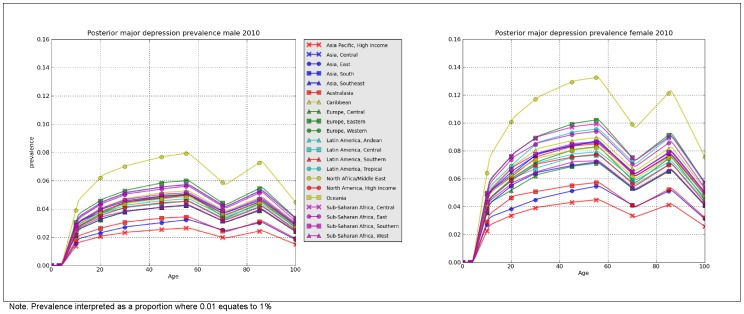
Regional point prevalence of MDD by age and sex, 2010. Presents the prevalence of MDD (as derived by DisMod-MR) by region, age and sex for 2010.

Figure 6 shows the final prevalence estimates by age, sex and region, for 2010. The equivalent data for 2005 and 1990 are summarised in supporting figures S1 and S2. When prevalence was aggregated by year (standardised by population age and sex [61]), the prevalence of MDD was very consistent between 1990 (4.4% (95% uncertainty: 4.2–4.7%)), 2005 (4.4% (4.1–4.7%) and 2010 (4.4% (4.1–4.7%)). Given the lack of time trend, the rest of the results will focus on the 2010 DisMod-MR output.

**Figure 7:**
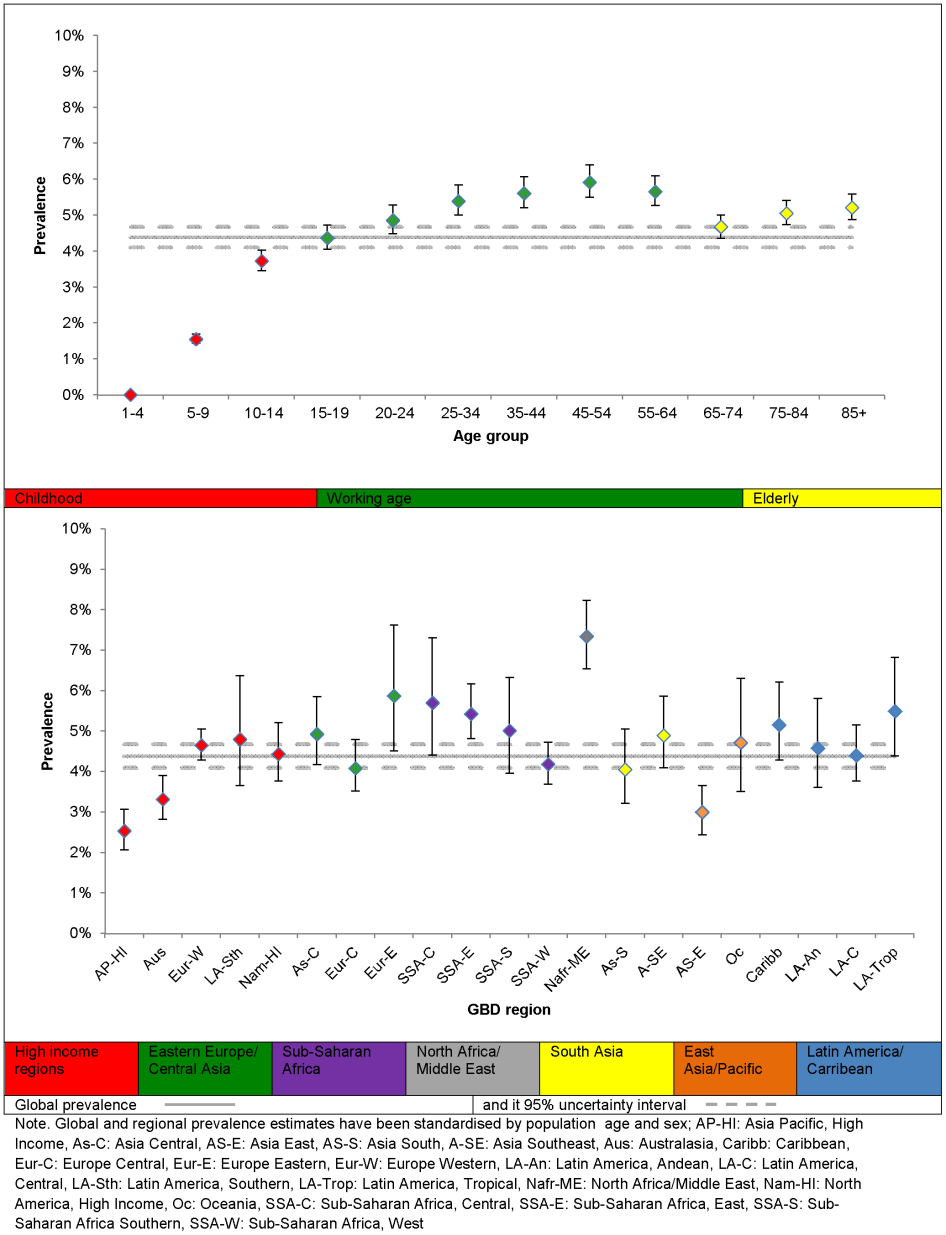
The overall point prevalence of MDD and 95% uncertainty by region and age, 2010. Presents the prevalence of MDD (as derived by DisMod-MR) by region and age for 2010.

Prevalence in 2010 was higher in females at 5.5% (5.0–6.0%) compared to males at 3.2% (3.0–3.6%), equivalent to a male: female prevalence ratio of 0.59 (0.54–0.64). When observed across the lifespan, prevalence increased steadily between 3 and 19 years; peaked between 20 and 64 years; decreased between 65 to 74 years; and showed a smaller increase in the oldest age group. Plot 1 in figure 7 summarises the age differences in the global prevalence of MDD. Note, for most age groups, estimates were within overlapping bounds of uncertainty. Plot 2 summarises the regional differences in the global prevalence of MDD. There was a 3-fold difference between North Africa/Middle East, the region with the highest prevalence and Asia Pacific, High income, the region with the lowest prevalence. Although this suggests considerable regional differences, the overlap in uncertainty intervals across regions is worth noting.

**Figure 8:**
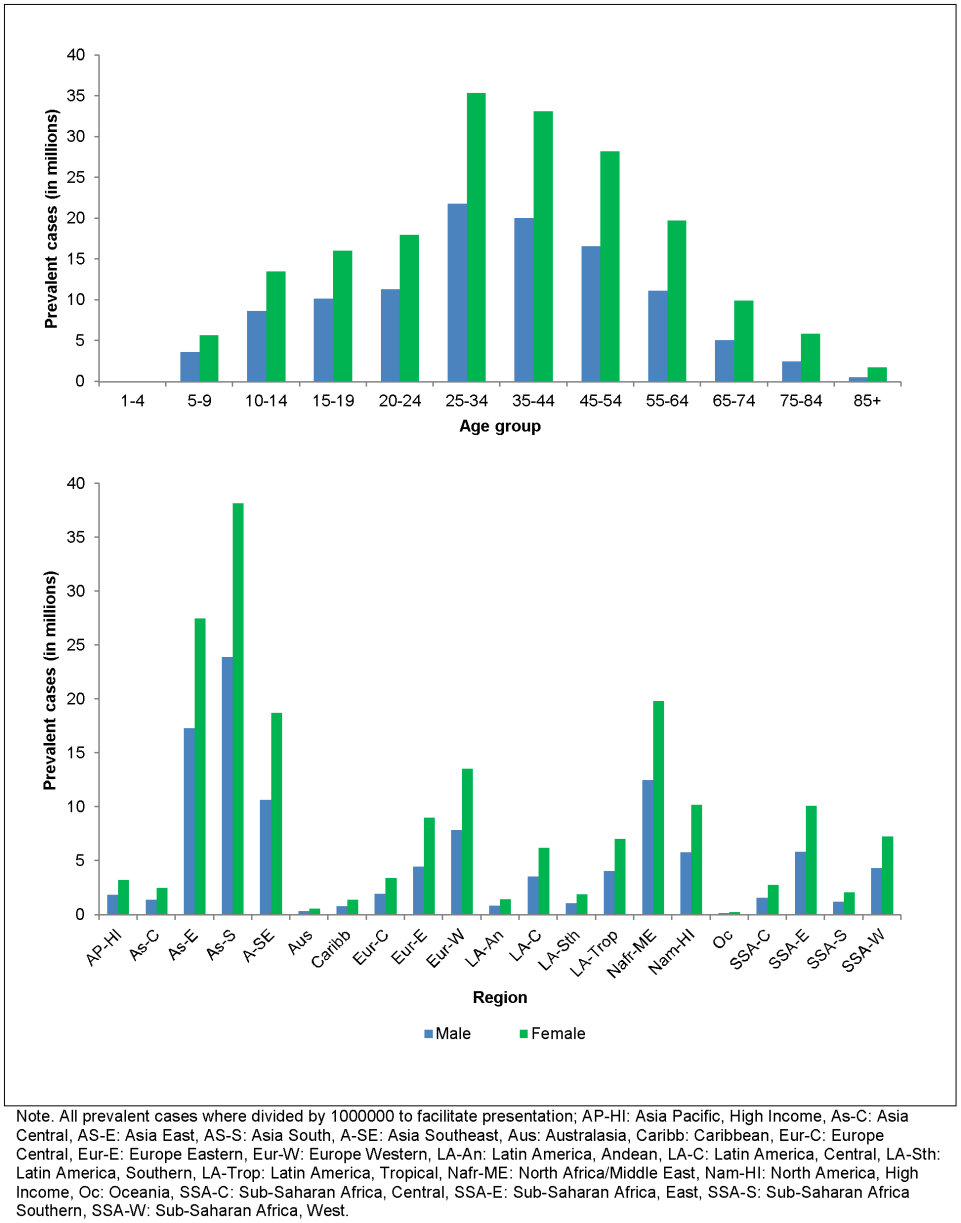
The number of point prevalent cases (in millions) of MDD by region, age and sex, 2010. Presents the prevalent cases of MDD (as derived by DisMod-MR) by region, age and sex for 2010.

When multiplied with United Nation's region-, sex-, year- and age-specific population size [62], the overall prevalence of MDD in 2010 corresponded to over 111 million male and 187 million female prevalent cases of MDD. The majority of cases appeared between 25 and 34 years at over 57 million cases and the least number of cases between 1 and 4 years at 19 thousand cases. Given their population size, Asia East and Asia-South yielded the highest number of prevalence cases at over 44 million and 62 million cases respectively. Prevalent cases by age and region have been summarised further in figure 8.

## Discussion

Consistent with previous reports, the prevalence of MDD (as estimated by DisMod-MR) was higher in females compared to males [63]–[65]. However, unlike our meta-regression outside of DisMod-MR which found that prevalence increased significantly over time [14], our findings differed; suggesting that the former could have been an artefact of measurement bias rather than a real difference in the disorder's epidemiology.

The pooled prevalence estimate derived by DisMod-MR was also more conservative than that from our meta-regression (4.4% (4.1–4.7%) vs., 4.7% (4.4–5.0%)). This difference was likely due to the differences in the age range for which estimates were pooled. In the meta-regression we aggregated data from different studies all using different age ranges (from 0–9 through to 65 to 99 years). DisMod-MR was much more versatile in this respect as it was able to aggregate estimates with different age ranges into the most plausible age pattern for the entire lifespan. This allowed us to more consistently measure differences in prevalence across the lifespan which was not possible in our analyses outside of DisMod-MR where we could only classify age using 4 broad age groups with some age ranges fitting into several groups. According to DisMod-MR findings, prevalence was lowest, but still evident in early childhood and highest between 20 and 64 years. There was an increase in prevalence between 75 and 85 years (5.1%(4.7–5.4%) and onwards (5.2%(4.9–5.6%), well within the prevalence range (4.6 to 9.3%) obtained by Luppa and collaborators' in their recent review and meta-analysis of the prevalence of MDD in those aged 75 years or above [66]. This age group is not always represented in population surveys as they tend to exclude people in residential care or non-private households [67]–[69]. Consequently, this finding has important implications for the burden calculation of MDD which as a result incorporated prevalent cases of MDD across the entire lifespan.

Also important for burden calculations was the ability to estimate prevalence (and therefore burden) for all 21 GBD regions, including regions like Oceania, Sub-Saharan-Africa Central, Latin America-Andean and Asia Central from where we had no empirical data. The regional pattern of prevalence was similar to that derived in our meta-regression [14], where prevalence from high income regions was lower than prevalence from low to middle income regions, particularly regions in conflict. Calculating the number of prevalent cases across world regions helps to emphasize the challenge confronting health services in responding to MDD. For instance, Asian regions which do not have the highest prevalence in comparison to other regions have the most prevalent cases due to their population size. That said, the low prevalence rate of MDD, in all Asian regions needs to be noted. Although the inclusion of depression NOS cases helped capture non-western presentations of MDD likely missed by DSM/ICD diagnostic criteria, it could be that it did not completely account for this limitation. This is especially true for studies where lay rather than clinically trained interviewers were used to diagnose cases [34]. Further investigation into the cross-cultural validity of DSM/ICD diagnostic criteria is required for clearer conclusions.

The process of modelling epidemiological parameters by GBD region necessarily dilutes the effects of conflict on prevalence of mental disorders which may otherwise be clearly demonstrated in country or local level surveys. Despite this, and combined with the fact that we found very few data points from populations in current or past conflict, we were still able to detect an increase in prevalence in those settings. Prevalence was highest in North Africa/Middle East which includes conflict countries such as Afghanistan, Iraq and Lebanon. This highlights the importance for future mental health research to provide comparable assessments of mental disorders in conflict-affected populations. The conflict covariates together with the survey instrument and prevalence type study-level covariates allowed us to accommodate for some, but not all of the variability in the epidemiological data available for MDD as DisMod-MR assumes the same level of adjustment for covariates across regions. The data presented here is reflective of the current state of this literature. With ongoing work on the global epidemiology and determinants of this disorder, we hope to explain more of the uncertainty around our final estimates.

With the emphasis on providing a ‘data driven’ epidemiological profile of MDD, we would have preferred to have incidence data inform our DisMod-MR output. Our search for data on the incidence of MDD revealed very low rates of MDD incidence in the population. An explanation for this is that the few longitudinal studies reporting on the incidence of MDD typically focused on capturing the incidence of MDD, rather than the incidence of major depressive episodes [53]–[56]. Given that MDD is being modelled as an episodic disorder for GBD, this means that new episodes in people with previous episodes were not counted and incidence was underestimated. By relying on prevalence, duration and excess-mortality data to calculate incidence, DisMod-MR derived incidence estimates which were higher than prevalence, illustrating much better internal consistency between the prevalence, incidence and duration output.

We used an average duration of 37.7 weeks which was higher than the 30.1 weeks reported by Vos and collaborators [24]. This difference was due to the inclusion of data from the Netherlands [49] previously excluded by Vos and collaborators on the basis that it included cases of subsyndromal depression and dysthymia. Given the lack of available duration data for all parts of the world except USA and that the median duration yielded by this study (12 weeks) was comparable to the median duration from other included studies (6–12 weeks) [45]–[48], we chose to include it. However, even with this inclusion, we did not have enough data to investigate and adjust for any age, sex and cross-national differences in the duration of a major depressive episode. This highlights the need for more studies following up community identified cases of MDD and measuring course of episode, particularly in low to middle income countries.

Rather than rely solely on sub-optimal estimates of prevalence, incidence, duration and excess mortality, we were able to model these into an internally consistent epidemiological profile of MDD. This will contribute to GBD 2010 and the MDD literature at large by providing global estimates for MDD which go beyond mere tabulation and pooling of epidemiological data. For some parameters, DisMod-MR had to rely on data from only a small number of studies, limiting the accuracy and generalisability of findings. This has been represented through large and at times, overlapping bounds of uncertainty which need to be considered while interpreting DisMod-MR estimates. As more evidence accumulates, the approach taken here will become increasingly sophisticated in its ability to synthesize available information and to project intelligent estimates into areas where data are not available.

## Supporting Information

Text S1 **PRISMA checklist and flowchart.** Summarises the PRISMA checklist and flowchart for the systematic review used to capture prevenance, incidence, duration, remission and excess mortality data.
(TIF)

Figure S1 **Regional point prevalence of MDD by age and sex, 1990.** Presents the prevalence of MDD (as derived by DisMod-MR) by region, age and sex for 1990.
(TIF)

Figure S2 **Regional point prevalence of MDD by age and sex, 2005.** Presents the prevalence of MDD (as derived by DisMod-MR) by region, age and sex for 2005.
(TIF)
