# Supplementary figures and images for: The Epidemiological Modelling of Major Depressive Disorder: Application for the Global Burden of Disease Study 2010
Source: PLoS One. 2013 Jul 29;8(7):e69637. doi: 10.1371/journal.pone.0069637 (PMC3726670; doi:10.1371/journal.pone.0069637)

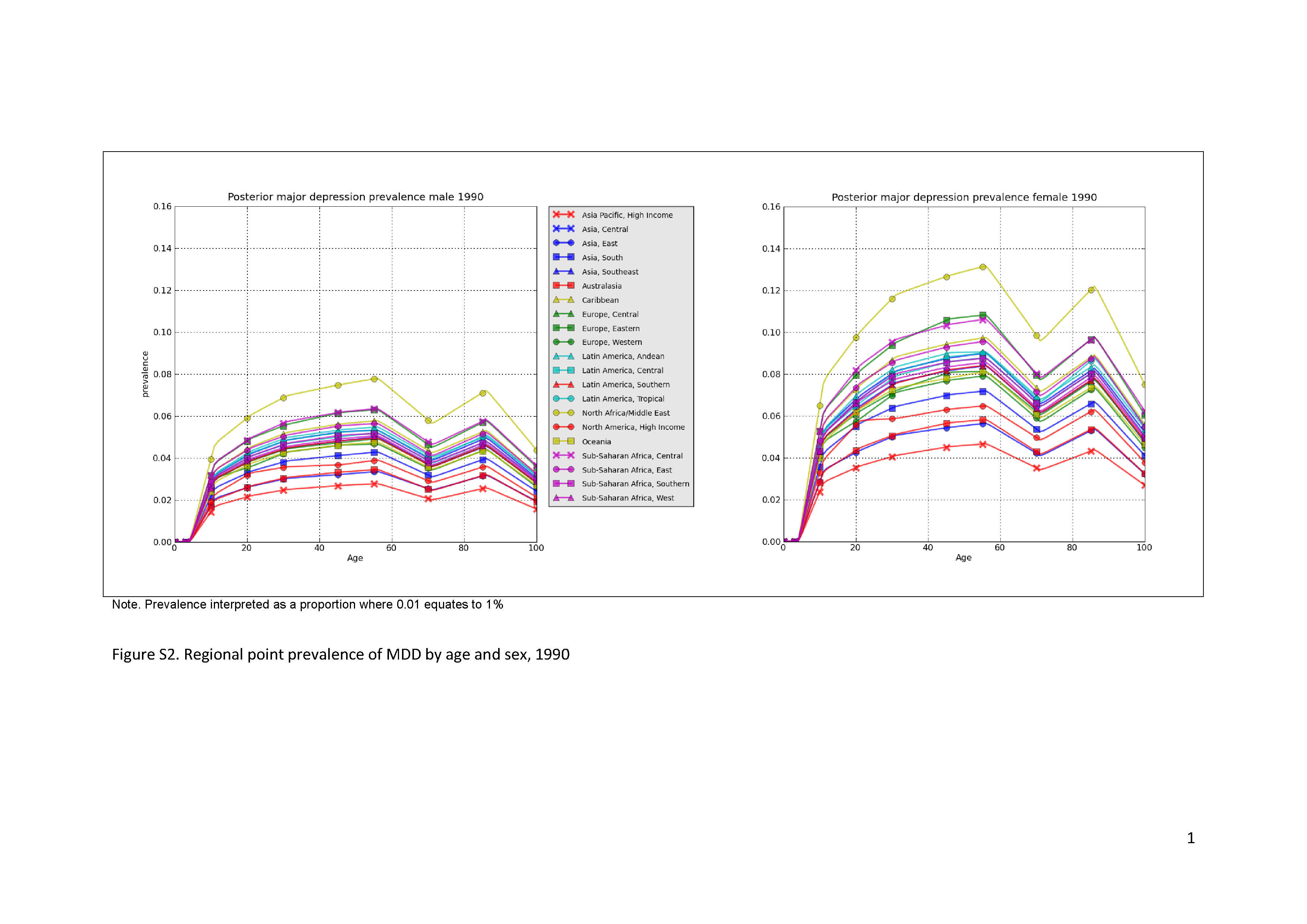

Supplement: Figure S1 — Regional point prevalence of MDD by age and sex, 1990. Presents the prevalence of MDD (as derived by DisMod-MR) by region, age and sex for 1990. (TIF) [file pone.0069637.s002.tif]

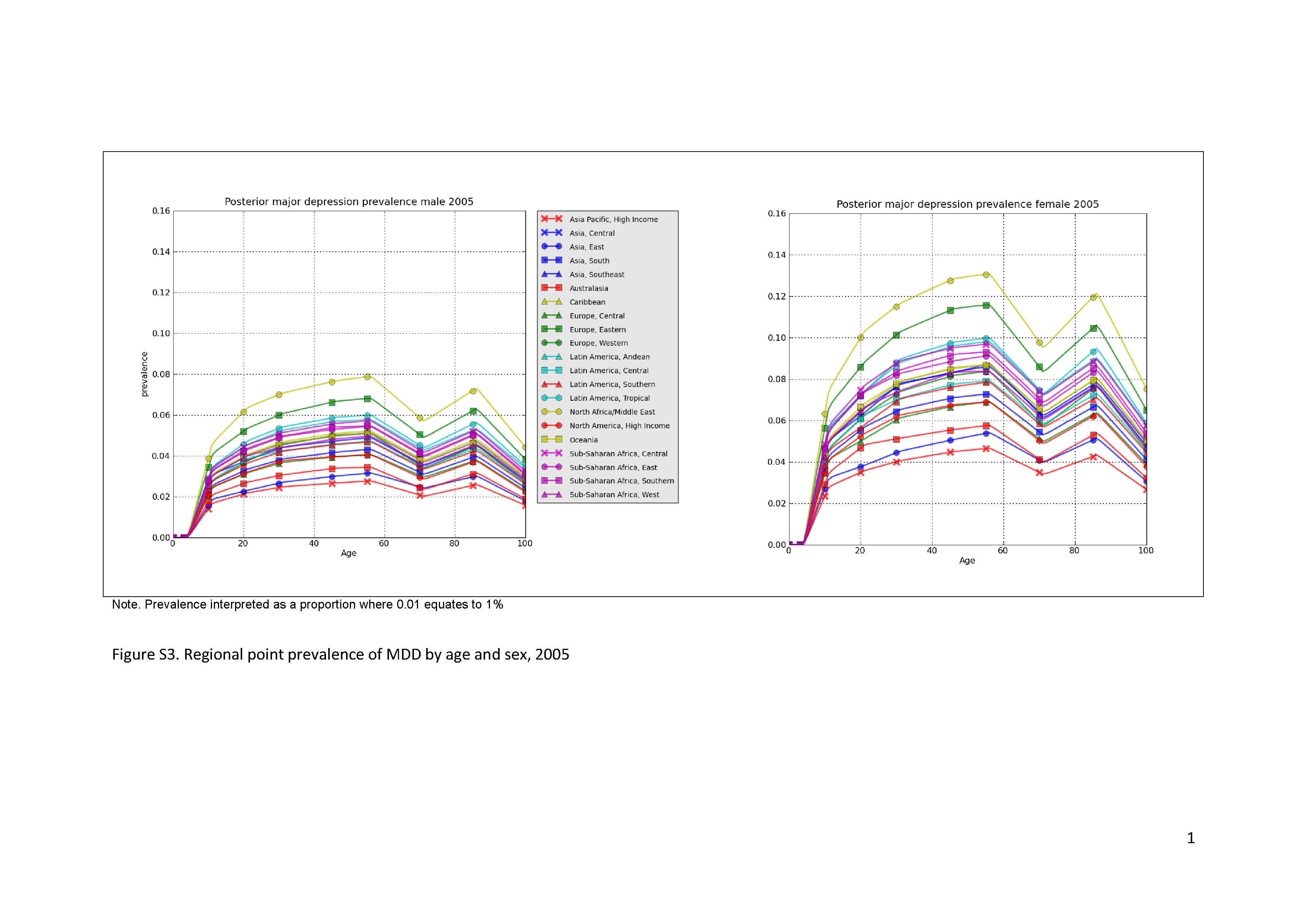

Supplement: Figure S2 — Regional point prevalence of MDD by age and sex, 2005. Presents the prevalence of MDD (as derived by DisMod-MR) by region, age and sex for 2005. (TIF) [file pone.0069637.s003.tif]
